# Enhanced Zone II Flexor Tendon Repair through a New Half Hitch Loop Suture Configuration

**DOI:** 10.1371/journal.pone.0153822

**Published:** 2016-04-21

**Authors:** Ioannis Kormpakis, Stephen W. Linderman, Stavros Thomopoulos, Richard H. Gelberman

**Affiliations:** 1 Department of Orthopaedic Surgery, Washington University, St. Louis, Missouri, United States of America; 2 Department of Biomedical Engineering, Washington University, St. Louis, Missouri, United States of America; 3 Department of Orthopedic Surgery, Columbia University, New York, New York, United States of America; 4 Department of Biomedical Engineering, Columbia University, New York, New York, United States of America; Mayo Clinic Minnesota, UNITED STATES

## Abstract

This study evaluated the impact of a new half hitch loop suture configuration on flexor tendon repair mechanics. Cadaver canine flexor digitorum profundus tendons were repaired with 4- or 8-strands, 4–0 or 3–0 suture, with and without half hitch loops. An additional group underwent repair with half hitch loops but without the terminal knot. Half hitch loops improved the strength of 8-strand repairs by 21% when 4–0, and 33% when 3–0 suture was used, and caused a shift in failure mode from suture pullout to suture breakage. 8-strand repairs with half hitch loops but without a terminal knot produced equivalent mechanical properties to those without half hitch loops but with a terminal knot. 4-strand repairs were limited by the strength of the suture in all groups and, as a result, the presence of half hitch loops did not alter the mechanical properties. Overall, half hitch loops improved repair mechanics, allowing failure strength to reach the full capability of suture strength. Improving the mechanical properties of flexor tendon repair with half hitch loops has the potential to reduce the postoperative risk of gap formation and catastrophic rupture in the early postoperative period.

## Introduction

Despite improved operative technique and postoperative rehabilitation, the outcomes of intrasynovial flexor tendon repair are highly variable [1–5]. The most commonly manifested complications of tendon repair are gap formation, repair-site failure, and adhesion formation between the repaired tendon and synovial sheath [6]. Typically, complications are noted within the initial few weeks following suture repair and depend, to a considerable extent, on the initial biomechanical properties of the repair [7]. Improving the biomechanical properties of repaired tendon is central to reducing the incidence of tendon rupture and gap formation, and for facilitating early controlled mobilization.

The initial mechanical properties of repaired tendon depend on the suture material and caliber and the interaction between the suture and tendon brought about by the repair technique [8–11]. Numerous suture materials and repair techniques have been described, including variations in core and peripheral suture caliber and purchase, numbers of strands and knot placement [10,12,13]. The most commonly used surgical techniques employ a 3–0 or 4–0 caliber core suture and a 5–0 or 6–0 caliber peripheral suture. Typically, either four, six, or eight strands are used and the final knot is placed either inside or outside the interface between the tendons stumps [13,14].

Although prior studies have demonstrated the importance of suture caliber and suture strand number on the initial mechanical properties of tendon repair [15], less consideration has been given to the mechanical effects created by the core suture loops. In the current study, our objective was to assess the mechanical interaction between tendon suture and Zone II flexor tendon (i.e., tendon grasping) as a function of the presence of a new style of half hitch loops (A.K.A. pretzel links), in an alternating configuration with the classic grasping loops, compared to repairs using grasping loops only. These half hitch loops form a small knot, gripping the tendon more than previously described locking loops [16,17]. Previous studies demonstrate that locking loops at every position lead to uneven tensioning and premature failure of the repair, motivating our alternating loop configuration [18]. To investigate this premise, we studied the effects of half hitch loops on 4- and 8-strand repairs performed with 3–0 and 4–0 suture. In addition, we considered a knotless configuration of the 8-strand repair using half hitch loops. Our hypothesis was that increased holding capacity between the tendon and the suture through the use of half hitch loops, in alternation with grasping loops, would improve the initial mechanical properties of 4- and 8-strand repair configurations.

## Materials and Methods

### Study design

In this *ex vivo* study, ninety-four cadaveric canine flexor digitorum profundus tendons were transected in Zone II, between the A2 and A4 pulleys. The tendons were divided into eight groups (Table 1) with either 4-strand modified Kessler or standard 8-strand repairs, with or without half hitch loops, using 3–0 or 4–0 suture. In addition, a group of tendons was repaired with an 8-strand knotless modification with half hitch loops. All repairs were performed by an orthopaedic hand surgeon. All tendons tested in this study were from hindpaws of healthy female adult mongrel dogs 20–30 kg in weight (Covance Research, Princeton, NJ), taken postmortem from an unrelated project. The unrelated project, which investigated experimental approaches for forepaw flexor digitorum profundus tendon repair *in vivo*, was approved by the Animal Studies Committee, Office of the Vice Chancellor for Research, Washington University in St. Louis for RHG’s Animal Approval Protocol #20140115. All work performed herein followed the policies and procedures for scientific research at Washington University in St. Louis, including the Research Integrity Policy by the Research Ethics and Compliance Office, Office of the Vice Chancellor for Research, Washington University in St. Louis.

**Table 1:** Flexor tendon suture configurations.

Technique	Strands	Caliber	N
Grasping	4	4–0	14
Hitched	4	4–0	12
Grasping	4	3–0	9
Hitched	4	3–0	9
Grasping	8	4–0	12
Hitched	8	4–0	12
Hitched knotless	8	4–0	12
Grasping	8	3–0	7
Hitched	8	3–0	7

### Half hitch loop technique

**Fig 1:**
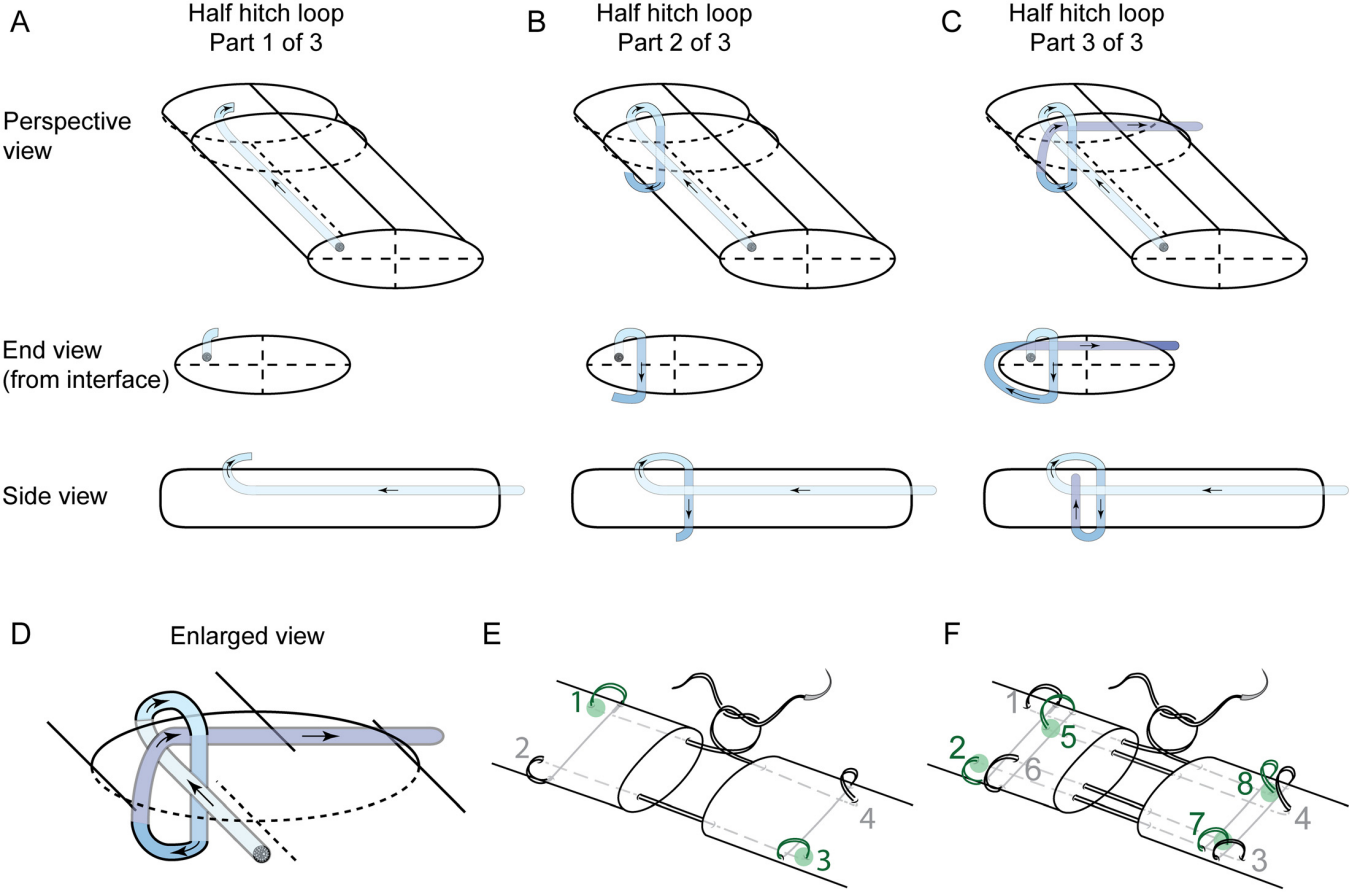
Suture configuration. **(A-C)** The three suture passes required to make a single half hitch loop are shown sequentially as an end view from the transection interface, a side view, and a perspective view. This is a continuous piece of suture, where suture coloration is artificially added to aid in following the suture path, going from blue to purple. The suture diameter is enlarged two-fold for clarity. Looped suture is used surgically, but only one of each pair of suture threads is diagrammed here for simplicity. **(D)** The complete half hitch loop configuration is enlarged for clarity. **(E-F)** The locations of half hitch loops are shown in green for 4-strand modified Kessler (E) and 8-strand Winters-Gelberman (F) repairs. In 8-strand repairs, the first four passes were performed on the dorsal side and the last four passes were performed on the ventral side of the tendon.

The technical modification introduced here shares some characteristics with the 4-strand modified Kessler and the 8-strand Winters-Gelberman repairs. A core suture purchase of 1.2 cm and a peripheral suture purchase of 2 mm was used (Fig 1). Supramid 4–0 or 3–0 looped double-strand suture (S. Jackson Inc., Alexandria, VA) was used for the core suture and 5–0 polypropylene was used for the peripheral suture. The repair consisted of half hitch and grasping loops of the core suture in an alternating fashion, which avoided the creation of consecutive hitches (Fig 1). This configuration is symmetric, with the same number of half hitch and grasping loops on the distal and proximal stumps, medially and laterally. This setup facilitated even tensioning of the repair, where each half hitch loop secured one stump while the adjacent grasping loop allowed gliding of the suture to occur. The sliding of one loop in each pair allowed flexibility for balancing the tension of the suture strands by the surgeon, avoiding uneven load bearing among the individual strands.

To perform the half hitch loop suture in a 4-strand repair, the first and third loops were tied (Fig 1e). To accomplish the half hitch loop configuration in an 8-strand repair, two additional alternating half hitch loops were created providing 4 hitches (second, fifth, seventh and eighth loops; Fig 1f). In addition, a knotless version of the 8-strand repair was performed in which the terminal knot was not tied and the free end of the suture was passed through the tendon substance using a free needle. The surgeon must carefully pass the sutures in the final step of each half hitch through the space created by the previous two passes in order to achieve a successful half hitch (Fig 1c and 1d).

### Biomechanical testing

Repairs were tested biomechanically using methods described previously [19,20]. The proximal tendon was gripped with a triangle-toothed grip and the distal phalanx was gripped in a custom grip. After preconditioning, repaired tendons were pulled in uniaxial tension until failure on a material testing machine (5866; Instron Corp., Norwood, MA). Failure mode (e.g., suture pullout or suture breakage) was recorded. The load to create a 2 mm gap (N), maximum load (N), extension at 20 N and at maximum load (mm), strain at 20 N and at maximum load (%), stiffness (N/mm) (the slope of the linear portion of the load–deformation curve), rigidity (N/%), and resilience (N strain) (the area under the load–strain curve up to the yield point) were determined using a custom-written code in MATLAB (Natick, MA), as described previously [21]. Load to create a gap of 2 mm between tendon stump ends (a threshold level that leads to decreased repair strength and increased adhesions) was calculated by optically tracking regions nearest to the repair site [4].

### Statistics

Groups were compared using an analysis of variance (ANOVA) followed by a Tukey's honest significant difference test for pairwise comparisons when appropriate. An alpha level of p < 0.05 was set for statistical significance. Results were plotted as mean ± standard deviations.

## Results

### Mechanical properties were improved when half hitch loops were incorporated into 8-strand repairs

**Fig 2:**
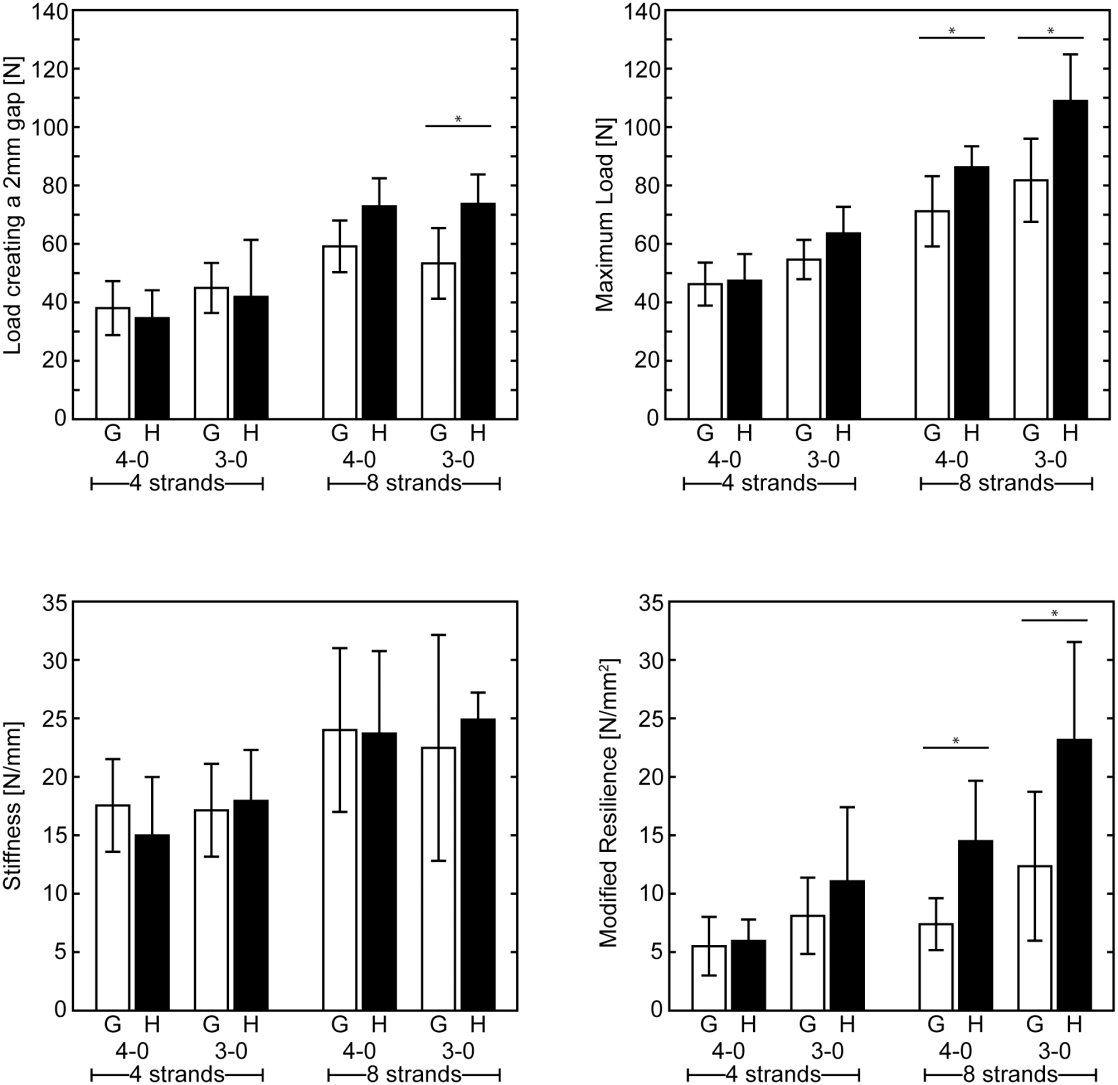
Mechanical properties. Load creating a 2 mm gap, maximum load, and resilience were significantly improved with the addition of half hitch loops in 8 strand repairs. (G: grasping, H: hitched, * p < 0.05, mean ± standard deviations are shown).

The addition of half hitch loops significantly improved the overall mechanical properties of 8-strand repairs. Load to create a 2 mm gap, maximum load, and resilience significantly increased in half hitch repairs compared to standard 8-strand repairs for 3–0 caliber suture, and maximum load and resilience significantly increased in half hitch repairs compared to standard 8-strand repairs for 4–0 caliber suture (p < 0.05 for each comparison; Fig 2). In contrast, the mechanical properties of the 4-strand repairs, with and without half hitch loops, were not significantly different (p > 0.05 for each comparison). Load to create a 2 mm gap, maximum load and resilience were not significantly different when comparing the modified 4-strand Kessler repair with and without half hitch loops, regardless of suture caliber (Fig 2). Stiffness was similar for all half hitch repair groups compared to their respective controls (Fig 2). 4-strand repairs with half hitch loops had statistically greater strain and extension to maximum load compared to modified Kessler repairs without half hitch loops (Table 2).

**Table 2:** Mechanical properties of repairs (mean ± standard deviation).

Technique	Strands	Caliber	Extension @ 20 N (mm)	Strain @ 20 N (%)	Extension @ Max Load (mm)	Strain @ Max Load (%)	Rigidity (N/%)
Grasping	4	4–0	1.56 ± 0.32	10.4 ± 2.2	5.08 ± 2.31	33.7 ± 15.3	2.64 ± 0.60
Hitched	4	4–0	1.59 ± 0.33	10.6 ± 2.2	7.32 ± 2.70	48.7 ± 17.9	2.25 ± 0.75
Grasping	4	3–0	1.74 ± 0.24	11.5 ± 1.5	7.50 ± 2.43	49.5 ± 15.8	2.59 ± 0.60
Hitched	4	3–0	1.54 ± 0.36	10.2 ± 2.4	7.43 ± 3.09	49.3 ± 20.5	2.71 ± 0.66
Grasping	8	4–0	1.33 ± 0.43	8.8 ± 2.9	5.51 ± 1.97	36.6 ± 13.2	3.62 ± 1.07
Hitched	8	4–0	1.60 ± 0.64	10.6 ± 4.3	6.88 ± 2.42	45.7 ± 16.1	3.57 ± 1.06
Hitched knotless	8	4–0	1.13 ± 0.14	7.5 ± 0.9	7.42 ± 2.11	49.0 ± 13.8	3.14 ± 0.65
Grasping	8	3–0	1.10 ± 0.96	7.3 ± 6.4	7.23 ± 3.50	48.2 ± 23.3	3.37 ± 1.46
Hitched	8	3–0	1.23 ± 0.11	8.2 ± 0.7	6.39 ± 1.19	42.4 ± 7.9	3.75 ± 0.35

### Failure mode shifted from suture pullout to suture breakage with the addition of half hitch loops

**Fig 3:**
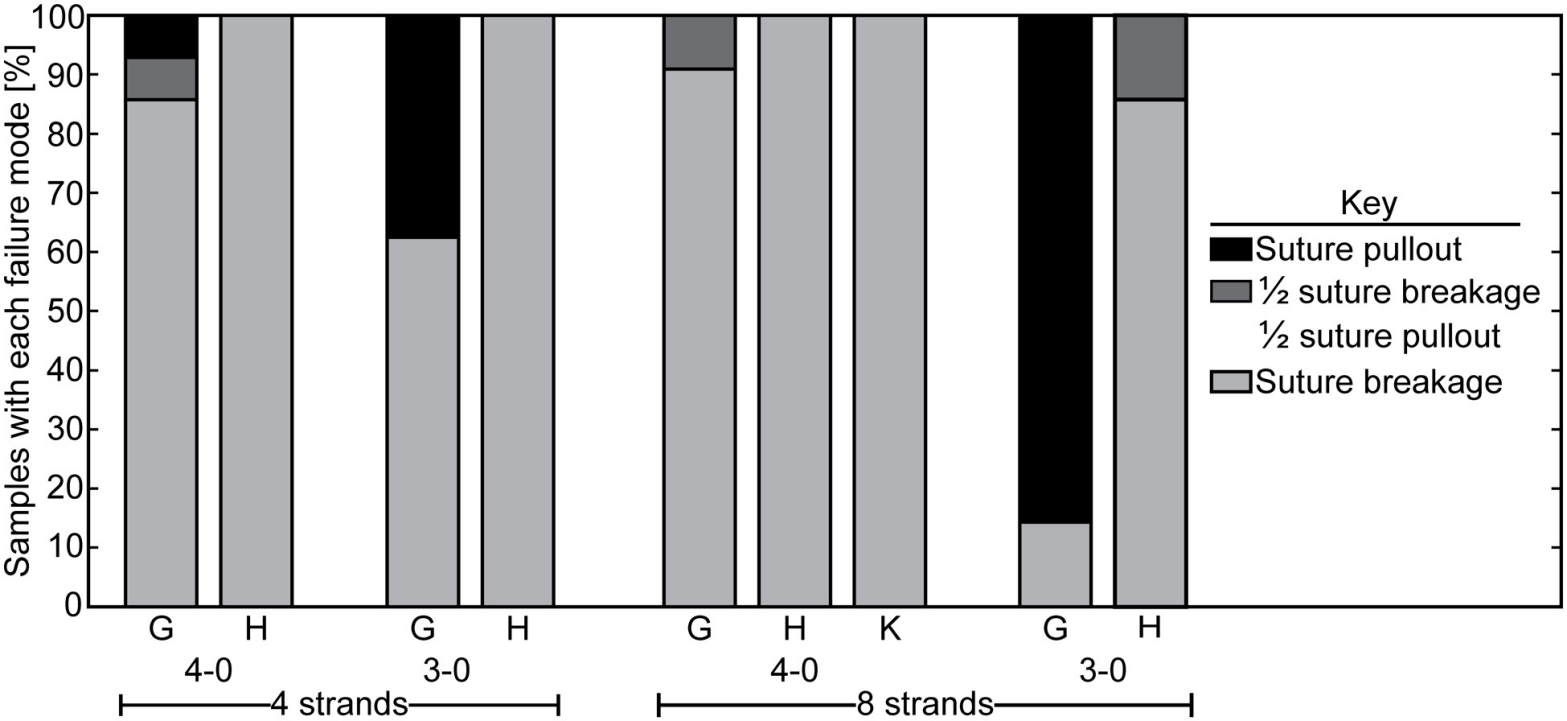
Failure modes. Repairs completed with 4–0 caliber suture failed almost exclusively by suture breakage. Increasing suture caliber from 4–0 to 3–0 led to a shift in failure mode from suture breakage to suture pullout. The addition of half hitch loops shifted the failure mode in 3–0 suture caliber repairs back to suture breakage, indicating a significant increase in suture-tendon interaction due to the hitches. (G: grasping, H: hitched, K: hitched knotless).

When suture caliber was increased from 4–0 to 3–0, the failure mode of the standard 8-strand repairs without half hitch loops shifted from suture breakage to suture pullout. When half hitch loops were added to the 8-strand repairs performed with 3–0 caliber sutures, the failure mode shifted from suture pullout to suture breakage, indicating improved tendon grasping by the suture due to the half hitch loops (Fig 3). The predominant failure mode of 4-strand repairs was suture breakage, regardless of suture caliber and the presence or absence of half hitch loops (Fig 3).

### The knotless configuration of the 8-strand repair with half hitch loops had equivalent mechanical properties to a standard 8-strand repair with a knot

**Fig 4:**
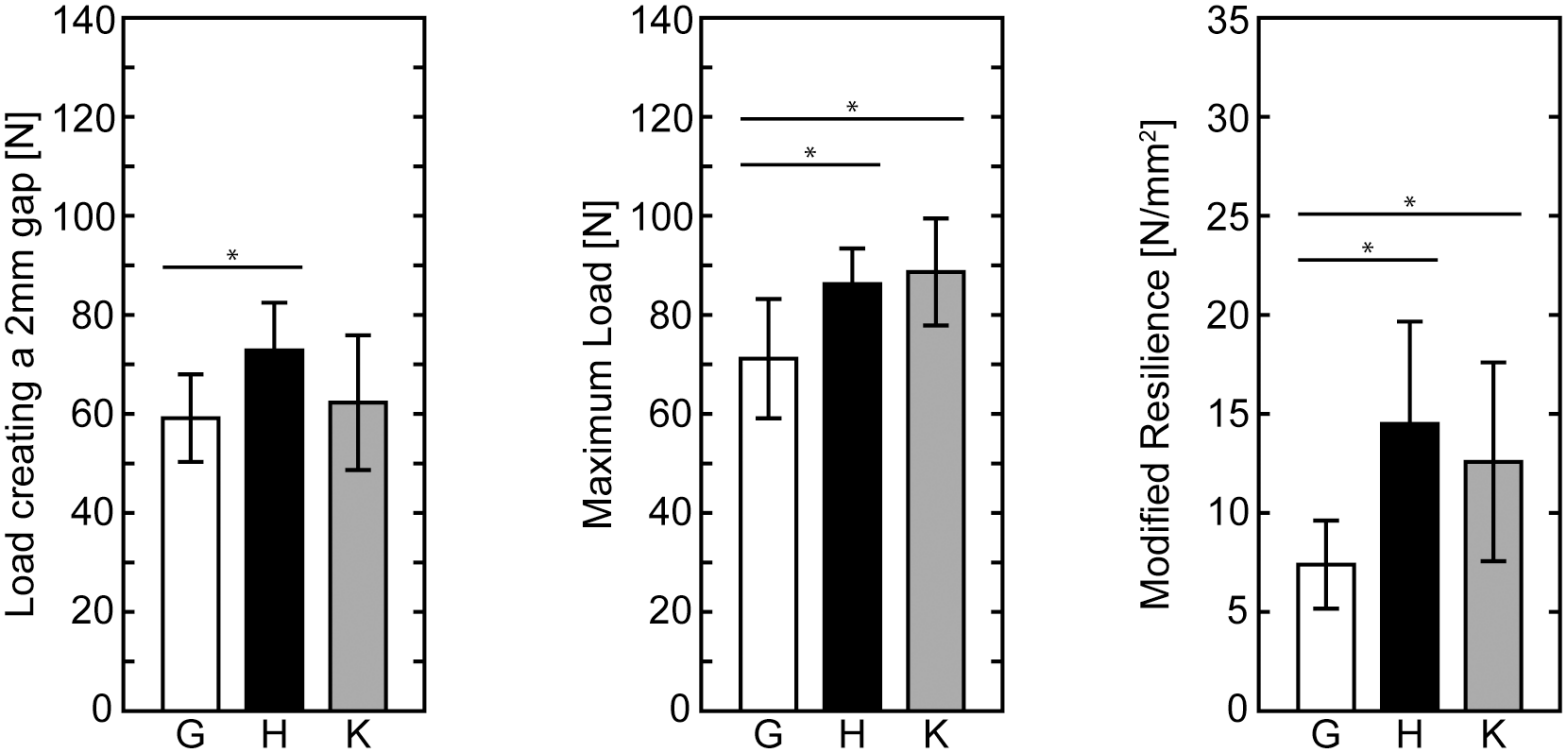
Mechanical properties of knotless repairs. Load creating a 2 mm gap, maximum load, and resilience significantly improved with the addition of half hitch loops. Knotless repairs were either equivalent (load creating a 2 mm gap) or improved (maximum load and resilience) compared to a standard Winters-Gelberman 8-strand repair (G: grasping, H: hitched, K: hitched knotless, * p < 0.05, mean ± standard deviations are shown).

The mechanical properties of half hitch loop repairs were similar regardless of whether or not the final knot between the tendon stumps was tied, indicating that the transfer of load occurred primarily at the half hitch loops, not at the final knot (Fig 4). Both knotted and knotless half hitch loop repair groups had significantly higher maximum load and resilience compared to the standard 8-strand repair (p < 0.05 for each comparison).

## Discussion

Prior studies have examined the effects of suture technique, suture material, and suture strand number on the time zero mechanical properties of flexor tendon repairs [9,15]. Previous studies investigating mechanical strength of various terminal knot tying methods show improved strength using reversing half hitches [22,23]. This common surgical knot tying technique was a motivation for testing a similar half hitch suture configuration applied to the suture loop instead of the terminal knot to improve tissue grasping at suture anchor points within the tendon. Few studies have explored the effects of increased suture-tendon interaction (i.e., improved tendon grasping) through the use of locking loops with the goal of improving mechanical properties of both 4- and 8-strand repairs. We are unaware of any previous studies investigating a similar half hitch loop for flexor tendon repair to the version described herein. In an *in vivo* study, Hatanaka et al. demonstrated that a locking suture configuration markedly enhanced ultimate strength compared to a grasping suture configuration three weeks post repair [24]. Similarly, another study showed that 3–0 caliber 4-strand cross-stitch locked repairs had significantly greater fatigue strength than did non-locked repairs and that the failure mechanism shifted from suture pullout to suture breakage [25]. On the other hand, Wong et al. showed that the consecutive locks in 6-strand repairs caused uneven load bearing among the strands, motivating the alternating half hitch and grasping loop approach described herein [18]. Employing a similar underlying principle, a recent study demonstrated that the addition of an adhesive coating to sutures in 8-strand repairs increased maximum load by 17% and load to create a 2 mm gap by 17.5% [21]. These results suggest that increasing the interaction between suture and the tendon, whether through suture half hitch loops or adhesives or a combination thereof, can lead to an improvement in the mechanical properties of the repair.

Findings in the current experimental study indicate that the failure of tendon repair depends on both the strength of the suture and its ability to grasp the tendon stumps. Increasing the number of strands and/or increasing suture caliber only increases tendon repair strength if the suture is effective in grasping tendon substance. If suture pulls out of tendon prior to breaking, the increased strength provided by additional suture material is not realized. An *ex vivo* study showed that cadaver flexor tendon repairs tend to fail by suture pullout, particularly when they are performed with 8-strands and higher caliber suture [15]. In our study, the utilization of half hitch loops in alternation with the grasping loops increased grasping strength in 8-strand repairs through more effective load transfer between the tendon and suture and through averting the uneven load distribution that results from consecutive locks or hitches. The effect was most profound when the repairs were performed with 3–0 suture, as the failure mode shifted from suture pullout to suture breakage. Furthermore, contrasting 8-strand repair groups indicated that the presence of an alternating half hitch loop configuration appeared to be of greater importance than an increase in suture caliber. Maximum load and load to 2 mm gap failed to increase significantly when increasing caliber from 4–0 to 3–0 suture. In contrast, 8-strand half hitch repairs performed with 4–0 suture yielded similar maximum load values and statistically greater load to 2mm gap compared to classic 8-strand repairs performed with 3–0 suture. These findings indicate that the use of this half hitch pattern has a greater influence on repair mechanics than does suture caliber.

Based on the improvements in repairs with half hitch loops, we explored a knotless technique and noted equivalent or improved mechanical properties compared to the standard Winters-Gelberman 8-strand repair. This result highlights the strength of the half hitch loops for transferring load across the repair site and could have significant implications for the *in vivo* setting. Instead of relying on the terminal knot, the half hitch loops held the suture in the appropriate configuration and prevented unraveling. Previous studies have examined the effects of knot placement on the mechanical properties of the repair and on adhesions formation during healing. Momose et al. demonstrated that placing the knot outside of the repair site improves tensile strength compared to placing the knot at the interface of tendons stumps (i.e., “inside” of the repair) [14]. However, this improvement in strength comes with a penalty: knot placement outside of the repair also increases tendon gliding resistance and tissue irritation, leading to adhesion formation. In order to minimize the effect of the knots, a number of knotless approaches have been introduced using barbed sutures, with mixed outcomes [26,27]. The knotless modification of the 8-strand repair shown here has the potential to combine the benefits to gliding and surgical operation time of a knotless repair with the benefits to tensile strength of the Winters-Gelberman repair technique.

While an improvement in mechanical properties was noted in the 8-strand repair group (increased load to create a 2 mm gap, failure load, and resilience), the mechanical properties of the 4-strand repairs was not significantly improved. We note that 4-strand repairs failed predominantly by suture breakage, even when 3–0 caliber suture was used. Therefore, the weakest component of the 4-strand repair appears to be the strength of the suture, not the tendon grasping strength.

There were several limitations to this study that may require further investigation. First, it is possible that the 8-strand half hitch repair may cause injury to the dorsal vascular supply of tendon due to the dorsal placement of some of the loops. However, dorsal placement has been shown to be well tolerated in prior *in vivo* studies at intervals through 3 and 6 weeks post repair [6,24]. Second, this study used canine cadaver tissue. Canine tendons may have different mechanical properties compared to human tendons. However, this animal model has been shown to have anatomic similarities to human tendons, in both *ex vivo* and *in vivo* experiments [28]. Third, we did not measure the gliding properties of the repairs in this *ex vivo* proof-of-concept study. The addition of half hitch loops to the repairs could affect gliding resistance, since more of the suture is laying on the tendon surface than in the traditional configurations. The similarity of the half hitch repairs to existing methods decreases the likelihood that gliding will be affected significantly. Future work should evaluate the work of flexion and gliding resistance prior to incorporating half hitch loops into clinical practice.

The results of this study confirm the hypothesis that increased tendon grasping strength through the use of half hitch loops, alternating with the grasping loops of the core suture, enhances the mechanical properties of 8-strand repairs, particularly when the repairs are carried out with 3–0 suture. The approach, however, did not enhance the properties of 4-strand repairs. Repair site failure mode appears to be of prime importance in predicting the mechanical properties of sutured intrasynovial tendon. In addition, the new half hitch loop configuration provided sufficient gripping capacity to allow for a knotless modification to the classical 8-strand repair. This knotless technique should be further evaluated *in vivo* for clinical utility.

## Supporting Information

S1 Dataset Data for each sample reported throughout this study. The first sheet provides the individual sample identifier, group type, failure mode, extension at 20 N, strain at 20 N, load to create a 2 mm gap, maximum load, extension at maximum load, strain at maximum load, stiffness, rigidity, and resilience up to the yield point. The second sheet summarizes these data as mean, standard deviation, and number of samples (n) for each sample group and result.
(XLSX)
